# Clinical analysis of 13 colorectal cancer patients with adrenal metastasis and a brief literature review

**DOI:** 10.1093/gastro/goae032

**Published:** 2024-05-02

**Authors:** Wei Chen, Shu-Yun Tan, Xiao-Qiong Chen, Xiao-Ping Tan, Jing-Lin Liang, Mei-Jin Huang

**Affiliations:** Department of General Surgery, The Sixth Affiliated Hospital, Sun Yat-sen University, Guangzhou, Guangdong, P. R. China; Guangdong Provincial Key Laboratory of Colorectal and Pelvic Floor Disease, The Sixth Affiliated Hospital, Sun Yat-sen University, Guangzhou, Guangdong, P. R. China; Guangdong Institute of Gastroenterology, The Sixth Affiliated Hospital, Sun Yat-sen University, Guangzhou, Guangdong, P. R. China; Department of General Surgery, The Sixth Affiliated Hospital, Sun Yat-sen University, Guangzhou, Guangdong, P. R. China; Guangdong Provincial Key Laboratory of Colorectal and Pelvic Floor Disease, The Sixth Affiliated Hospital, Sun Yat-sen University, Guangzhou, Guangdong, P. R. China; Guangdong Institute of Gastroenterology, The Sixth Affiliated Hospital, Sun Yat-sen University, Guangzhou, Guangdong, P. R. China; Department of General Surgery, The Sixth Affiliated Hospital, Sun Yat-sen University, Guangzhou, Guangdong, P. R. China; Guangdong Provincial Key Laboratory of Colorectal and Pelvic Floor Disease, The Sixth Affiliated Hospital, Sun Yat-sen University, Guangzhou, Guangdong, P. R. China; Guangdong Institute of Gastroenterology, The Sixth Affiliated Hospital, Sun Yat-sen University, Guangzhou, Guangdong, P. R. China; Department of Emergency, The Second Affiliated Hospital of Guangzhou Medical University, Guangzhou, Guangdong, P. R. China; Department of General Surgery, The Sixth Affiliated Hospital, Sun Yat-sen University, Guangzhou, Guangdong, P. R. China; Guangdong Provincial Key Laboratory of Colorectal and Pelvic Floor Disease, The Sixth Affiliated Hospital, Sun Yat-sen University, Guangzhou, Guangdong, P. R. China; Guangdong Institute of Gastroenterology, The Sixth Affiliated Hospital, Sun Yat-sen University, Guangzhou, Guangdong, P. R. China; Department of General Surgery, The Sixth Affiliated Hospital, Sun Yat-sen University, Guangzhou, Guangdong, P. R. China; Guangdong Provincial Key Laboratory of Colorectal and Pelvic Floor Disease, The Sixth Affiliated Hospital, Sun Yat-sen University, Guangzhou, Guangdong, P. R. China; Guangdong Institute of Gastroenterology, The Sixth Affiliated Hospital, Sun Yat-sen University, Guangzhou, Guangdong, P. R. China

## Background

With global lifestyle changes and economic development, the incidence of colorectal cancer (CRC) has been increasing annually. However, its survival rate is clearly less satisfactory [1]. Extra-intestinal metastasis has had an increasingly important impact on patient prognosis. The adrenal gland is one of the most common organs involved in CRC metastasis and the incidence of adrenal metastasis is 1.9%–17.4% [2]. The primary pathway for adrenal metastasis from CRC is hematogenous spread, but it can also occur through lymphatic spread or local invasion. Synchronous metastasis is diagnosed when it is observed as being present at the time of CRC diagnosis; complete metastasis after intestinal resection is termed metachronous metastasis. Unilateral metastasis is more common on the right side than on the left side, although bilateral metastasis can also occur. Patients with synchronous solitary adrenal metastasis have a worse prognosis than do those with metachronous solitary adrenal metastasis. However, in both scenarios, surgery should be considered, as some patients who undergo adrenal gland removal have a favorable long-term prognosis. Due to the non-specific clinical manifestations of adrenal metastasis, many patients may not exhibit relevant symptoms, which often prevents them from drawing the attention of clinicians. With advancements in imaging technology and improved postoperative follow-up of tumor patients, hospitals at all levels have seen an increasing detection rate for adrenal metastasis in CRC patients in recent years.

At present, there is still considerable debate regarding the treatment of adrenal metastasis from CRC. Early detection and diagnosis are difficult, and there is no unified conclusion on the selection of examination and treatment methods. This has led to certain problems in actual clinical practice. To investigate the surgical treatment outcomes of patients with CRC and adrenal metastasis, we conducted a summary analysis of the data from 13 patients.

## Patients and methods

Between January 2011 and May 2023, we screened 24,499 CRC patients who underwent surgery at the Sixth Affiliated Hospital of Sun Yat-sen University (Guangzhou, China). Patients who were confirmed to have a single adrenal metastasis during initial diagnosis or follow-up were examined and their clinical data were collected. All patients underwent resection of both of the CRC and adrenal gland. According to the size of the adrenal metastasis, body mass index, degree of periadrenal fat adhesion, and surgical history, different surgical approaches were selected. Patients with failed follow-up, incomplete data, or other malignant tumors were excluded. Ultimately, 13 eligible patients were included in the study. The examinations included enhanced computed tomography (CT) scans of the chest and abdomen, and functional tests of the adrenal cortex and medulla. All patients were in good physical condition (ECOG 0–1) and provided informed consent. The study was approved by the Institute Research Medical Ethics Committee of Sun Yat-sen University.

All patients were followed up until 30 May 2023. Survival time was calculated in months. The end-point for tumor-free survival was the time of first postoperative imaging confirmation of tumor recurrence or metastasis through methods such as CT, ultrasound, or magnetic resonance imaging. The overall survival time was calculated from the date of surgery to the date of death or the last follow-up.

## Results

There were nine males and four females, with a median age of 56 (33–69) years. All patients underwent surgical treatment (laparoscopic surgery in eight patients and open surgery in five patients). Moreover, there were 13 cases of unilateral adrenal metastasis (3 on the left side and 10 on the right side). The shortest time interval between the diagnosis of primary CRC and the occurrence of adrenal metastasis was 3.4 months, while the longest was 69.3 months. Simultaneous and metachronous adrenal metastases from CRC occurred in 2 and 11 patients, respectively. No patients had manifestations of Cushing’s syndrome or adrenocortical insufficiency.

Preoperative Hb, albumin, CA125, CA-153, CA199, CEA, and alpha-fetoprotein (AFP) levels were 118.07 ± 5.31 g/L, 39.38 ± 5.31 g/L, 47.46 ± 119.43 U/mL, 8.77 ± 3.77 U/mL, 35.41 ± 47.64 U/mL, 18.51 ± 28.58 ng/mL, and 3.16 ± 1.29 ng/mL, respectively. In addition, the surgical blood loss, operation time, and length of hospital stay were 228.46 ± 205.53 mL, 273.46 ± 85.42 min, and 19.23 ± 8.31 days, respectively. The postoperative overall survival (OS) time of patients ranged from 1.8 to 103.9 months, with a median survival time of 38.2 months. Three patients died due to tumor recurrence and the other patients were still alive. All the patients underwent resection for CRC with adrenal metastasis; the characteristics of the tumors are listed in Table 1.

**Table 1.** Characteristics of the patients with resected colorectal cancer who underwent adrenalectomy

No.	TNM	Interval time of adrenal metastasis	DFI (months)	Size (cm)	Adrenalectomy	Blood loss (mL)	Adjuvant therapy after adrenalectomy	Status	Recurrence	Survival after adrenalectomy (months)	ctDNA	IHC
1	T3N2M1	Sim	13.0	2.7 × 1.7	Laparoscopy	100	Chemotherapy	Dead	Yes	59.1	–	CK20 (+), CDX2 (+), Villin (+)
2	T3N2M0	Met	4.7	3.4 × 3.3	Laparotomy	200	Chemotherapy	Alive	No	89.8	–	CK7 (–), CK20 (+), CDX2 (+)
3	T3N2M0	Met	23.9	1.8 × 1.1	Laparotomy	400	Chemotherapy	Dead	Yes	20.3	–	CK20 (+), CDX2 (+), CK7 (–)
4	T3N1M0	Met	3.4	2.8 × 1.6	Laparoscopy	300	Chemotherapy	Alive	No	103.9	–	CK20 (+), CDX2 (+), Villin (+)
5	T3N2M0	Met	69.3	3.5 × 3.0	Laparotomy	700	Chemotherapy	Dead	Yes	29.9	EGFR, TP53	CK20 (+), CK7 (–), CDX2 (+)
6	T3N0M0	Met	24.7	4.1 × 3.2	Laparoscopy	50	Chemotherapy	Alive	No	28.4	–	CK20 (–), CK7 (–), CDX2 (+), PAX8 (–)
7	T3N0M0	Met	38.7	2.0 × 2.2	Laparoscopy	10	Chemotherapy	Alive	No	2.3	N	CK20 (+), CK7 (–), CDX2 (+)
8	T3N0M1	Sim	43.8	1.7 × 2.8	Laparoscopy	50	Chemotherapy	Alive	No	43.8	N	CK20 (+), CK7 (+), CDX2 (+)
9	T3N0M0	Met	13.8	6.1 × 5.4	Laparotomy	300	Chemotherapy	Alive	Yes	38.2	N	CK20 (+), CDX2 (+)
10	T4N1M0	Met	4.2	1.4 × 1.4	Laparoscopy	300	Chemotherapy	Alive	No	43.4	TP53	CK20 (+), CK7 (–), CDX2 (+)
11	T4N0M0	Met	31.8	1.3 × 1.0	Laparotomy	500	Chemotherapy	Alive	Yes	1.8	Kras, TP53, PIK3CA, BRAF	–
12	T3N0M0	Met	35.4	2.0 × 1.1	Laparoscopy	50	Chemotherapy	Alive	Yes	49.3	–	CK20 (+), CK7 (–), CDX2 (+)
13	T3N1M1	Met	13.6	2.8 × 3.2	Laparoscopy	10	Without chemotherapy	Alive	Yes	3.5	Kras, APC	CK20 (+), CK7 (–), CDX2 (+)

DFI = disease-free interval, Met = metachronous, Sim = simultaneous, IHC = immunohistochemistry.

## Discussion

In recent years, the incidence of adrenal metastasis cancer has been on the rise and the most common primary sites are the lung, breast, and kidney. Early diagnosis and differential diagnosis of this disease are still difficult. However, whether, when, and how to surgically remove adrenal metastases remain controversial. Adrenal metastases are often associated with systemic spread of disease and are considered to indicate unsuitability for surgical resection. However, aggressive surgical resection of adrenal metastases has been reported to improve OS [3].

Multiorgan metastasis is a key characteristic of advanced CRC metastasis. The adrenal gland is one of the main organs affected by hematogenous metastasis in late-stage CRC. Due to the concealed location and non-specific early symptoms, adrenal metastasis is often overlooked. Ultrasound, CT, and positron emission tomography–CT are important diagnostic methods for detecting adrenal metastasis in CRC patients, with sensitivities reaching 66%, 81%, and 100%, respectively [4]. Patients with adrenal metastasis from CRC are often perceived to have tumors that have metastasized throughout the body, indicating a poor prognosis. Whether the removal of metastatic adrenal cancer can change the natural course of stage IV tumors has been a topic of debate. Although the surgical removal of metastatic CRC in the adrenal gland has not yet been widely practiced, surgical removal is being adopted in more patients, especially those with lung cancer and renal cell carcinoma. Recent research showed that adrenalectomy was an independent predictor of favorable survival in 14 patients with adrenal metastases from isochronous CRC, with 3 patients experiencing recurrence within 1 year of surgery and 6 patients surviving for >4 years after surgery [5]. Similarly, in our investigation, all of patients with CRC adrenal metastasis had a median survival time of 38.2 months after complete removal of the metastatic cancer; among the 13 patients, 3 survived for nearly 5 years. Patients who underwent surgical treatment had better survival than those who received nonsurgical treatments for adrenal metastasis [6]. Therefore, we believe that, in patients who have a single site of metastasis along with primary cancer that is under control and who are physically amenable to enduring surgery, surgical treatment can lead to long-term benefits.

Open surgery and laparoscopic surgery are effective methods for treating tumors in the adrenal region. Compared with open surgery, laparoscopic surgery has obvious advantages in terms of operation time, intraoperative blood loss, and postoperative recovery. However, whether laparoscopic surgery is suitable for primary or metastatic adrenal malignancies remains controversial. After Elashry *et al*. [7] first reported two patients with adrenal malignant tumors undergoing laparoscopic adrenalectomy, other published reports followed, and the results were similar to those for open surgery [8]. In this group of patients, we performed laparoscopic surgery on eight patients and laparotomy on five patients. We found that laparoscopic surgery for small adrenal metastases also offers good safety and sufficient surgical capacity. Preparation and careful and gentle intraoperative manipulation are also key to successful surgery. In addition, the efficacy of laparoscopic surgery for the treatment of malignant adrenal tumors is affected by factors such as the operator's technical proficiency, case selection, and whether standard radical surgery is performed. Therefore, many cases need to be verified over a long period of time.

Based on our experience, preoperative imaging confirmed that the metastatic cancer was confined within the adrenal capsule, with no local lymph node enlargement and no extrarenal metastasis, suggesting that patients can undergo adrenal removal to treat the adrenal metastatic cancer. Furthermore, in patients with primary tumors that can no longer be completely removed or those with multiple metastases, surgical opportunities are lost. It is thus essential to opt for radiation, chemotherapy, or immunotherapy. Improving the quality of life for patients can be accomplished through appropriate palliative care. With the continuous development and advancement of minimally invasive tumor treatment techniques, various ablation treatments and radiotherapies have been widely used in tumor treatment. These methods have enriched the treatment options available for patients with metastatic adrenal cancer and effectively improved the treatment results [9].

## Conclusions

Solitary adrenal metastasis derived from CRC is rare. When the primary CRC can be resected or well controlled and there is no other evidence of metastasis, adrenalectomy is recommended.

## Authors’ Contributions

W.C., X.Q.C.: study design, data collection, data analysis and interpretation, draft of the manuscript, approval of final manuscript, supervision. X.P.T., S.Y.T.: study design, data collection, data analysis and interpretation, draft of the manuscript, approval of final manuscript, supervision. J.L.L.: study design, data analysis and interpretation, revision of the manuscript, approval of final manuscript. M.J.H.: study design, data collection, data analysis and interpretation, revision of the manuscript, approval of final manuscript. All authors approved the final version of the manuscript.

## Data Availability

The data used and/or analysed during the current study are available from the corresponding author on reasonable request.
